# Prognostic Value of Preoperative Nutritional Assessment and Neutrophil-to-Lymphocyte Ratio in Patients With Thymic Epithelial Tumors

**DOI:** 10.3389/fnut.2022.868336

**Published:** 2022-07-08

**Authors:** Yang-Yu Huang, Shen-Hua Liang, Yu Hu, Xuan Liu, Guo-Wei Ma

**Affiliations:** State Key Laboratory of Oncology in South China, Collaborative Innovation Center for Cancer Medicine, Sun Yat-sen University Cancer Center, Guangzhou, China

**Keywords:** thymic epithelial tumor, prognostic factor, nutritional risk index, neutrophil-to-lymphocyte ratio, overall survival, recurrence free survival

## Abstract

**Introduction:**

Systemic nutrition and immune inflammation are the key factors in cancer development and metastasis. This study aimed to compare and assess four nutritional status and immune indicators: prognostic nutritional index (PNI), nutritional risk index (NRI), neutrophil-to-lymphocyte ratio (NLR), and the systemic immune-inflammatory index (SII) as prognostic indicators for patients with thymic epithelial tumors.

**Materials:**

We retrospectively reviewed 154 patients who underwent thymic epithelial tumor resection at our hospital between 2004 and 2015. The optimal cutoff value for each nutritional and immune index was obtained using the X-tile software. Kaplan-Meier curves and Cox proportional hazards models were used for survival analysis.

**Results:**

Univariate analysis showed that PNI, NRI, NLR, SII, albumin (ALB), the albumin/globulin ratio (A/G), WHO stage, T stage, and drinking history were associated with the overall survival (OS) of patients (*P* < 0.05). The NRI, NLR, A/G, ALB, T stage, and WHO stage were significant independent prognostic factors of OS in multivariate analysis (*P* < 0.05). Finally, we constructed a coNRI-NLR model to predict OS and recurrence-free survival (RFS).

**Conclusions:**

This study suggests that the preoperative NRI, NLR, and coNRI-NLR model may be important prognostic factors for patients with thymic epithelial tumors who undergo surgical resection.

## Introduction

Thymic epithelial tumors are rare malignancies which frequently occur in the anterior mediastinum of adults, and include thymomas and thymic carcinomas (1, 2). Although surgery is an effective treatment, since thymic epithelial tumors only account for around 0.2–1.5% of all malignancies, there is currently no standard, comprehensive treatment protocol (3, 4). A recent meta-analysis showed that postoperative radiotherapy can improve the overall survival rate of Masaoka-Koga stage II and III thymoma, but no prospective studies have confirmed these results (5). Additionally, a study of postoperative chemotherapy has not yet reached a definite conclusion, because thymomas are an indolent tumor with a low incidence and relatively long survival time. Therefore, it is difficult to predict tumor prognosis and recurrence and to formulate individualized treatment plans.

Preoperative nutritional status is associated with postoperative complications and overall survival (OS) in patients with cancer (6), and many indicators containing nutritional variables have been found to play a role in predicting the prognosis of patients with various cancer, such as esophageal cancer (7), non-small cell lung cancer (8), colorectal cancer (9), and oral cancer (10). However, the relationship between the nutritional risk index (NRI) or prognostic nutritional index (PNI) and clinical outcomes in patients with thymic epithelial tumors remains unclear and has not been validated.

Additionally, inflammation plays an important role in the development and progression of cancer (11–13). Inflammation-related indicators such as the systemic immune-inflammatory index (SII) and neutrophil-to-lymphocyte ratio (NLR) play a role in predicting prognosis in breast cancer (14), kidney cancer (15), lung cancer (16), esophageal cancer (17, 18), and other tumors. Considering the close relationship between inflammation and tumor development, this study also assessed inflammation-related factors.

As research predicting tumor prognosis and recurrence is of great significance when determining individualized treatment and postoperative adjuvant therapy for patients with thymic epithelial tumors, we studied the ability of the four most commonly reported nutritional and immune-inflammation-related indicators (PNI, NRI, SII, and NLR) to predict the prognosis of thymic epithelial tumors. In addition, we explored new indicators that have an impact on prognosis, in order to more accurately and conveniently predict the prognosis and recurrence of thymic epithelial tumors.

## Materials and Methods

This study was approved by the Medical Ethics Committee of Sun Yat-sen University Cancer Center (B2020-353-01), and included patient data collected at Sun Yat-sen University Cancer Center (record number: RDDA2021002090). The study complied with the Declaration of Helsinki.

This study retrospectively analyzed 154 patients who underwent thymic epithelial tumor resection at our center between May 2004 and August 2015. The inclusion criteria were as follows: (1) age > 18 years; (2) complete surgical resection (R0, no residual disease); (3) presence of histopathologically confirmed thymic epithelial tumors, including thymoma and thymic carcinoma (TC); and (4) complete relevant laboratory tests (such as routine blood tests, and routine biochemical tests) within 7 days before surgery. The exclusion criteria were as follows: (1) Patients who received radiotherapy or chemotherapy prior to surgery, before and after surgery, or an unknown sequence of treatment with surgery. (2) Patients with more than one malignancy or history of other malignancies. (3) Postoperative survival time less than 3 months. (4) Follow-up time less than 5 years. (5) Cryoablation as the surgical method. (6) If the patient only underwent thymoma biopsy. (7) Incomplete follow-up information.

### Data Collection

Data were collected for the following clinical variables: hematological indicators (obtained within 1 week before surgery), lymphocyte count, neutrophil count, albumin level (ALB), platelet count, globulin level, patient's age, sex, smoking history, drinking history (drinking alcohol every day, although the specific amount of drinking was not limited or described), family history of tumors, tumor size, myasthenia gravis symptoms, histological subtype, and body mass index (BMI). In this study, T staging was obtained by combining imaging data with intraoperative records and postoperative pathological information, and we staged all patients according to the 8th edition of the TNM staging system.

### Follow Up

Patients were followed-up every 6–12 months for the first 2 years, every 12 months for the third to fifth years, and annually thereafter. The follow-up investigations included chest CT scan and hematological examination (including routine blood tests, routine biochemical tests, and investigation of tumor markers), and the final follow-up timepoint was August 2020. The primary endpoints were overall survival (OS) and recurrence free survival (RFS).

### Variable Definition

All hematological indicators were collected within 7 days before surgery. The formula for calculating nutritional indicators is as follows: BMI = weight/height^2^ (kg/m^2^); NLR = neutrophil count/lymphocyte count; SII = platelet count × neutrophil count/lymphocyte count; PLR = platelet count/lymphocyte count; PNI = albumin (g/l) + 0.005 × lymphocyte count (μl), as derived from Onodera et al. (19). NRI was calculated according to the formula: NRI = (1.519 × albumin, g/1) (41.7 × current/ideal body weight), as defined by Buzby et al. (20). The ideal body weight was calculated according to the Lorenz equation; for males: Height – 100 – [(Height – 150)/4], and for females: Height – 100 – [(Height – 150)/2.5].

### Data Analysis

Statistical analyses were performed using SPSS 25.0 (IBM, Chicago, Illinois, USA), and R software (version 4.0.3; https://www.r-project.org/). X-Tile software was used to obtain the optimal cutoff values for nutritional and inflammatory predictors (http://www.tissuearray.org/rimmlab). Survival analysis was performed using the Kaplan-Meier log-rank test. Univariate and multivariate analyses were performed using Cox proportional hazard regression models. Relative risks were assessed using hazard ratios (HRs) and 95% confidence intervals (CI). Receiver operating characteristic (ROC) curve analysis was used to compare area under the curve (AUC) values between different models. All tests were two-way, and the significance level was set at *p* < 0.05.

## Results

### Patient Characteristics

A total of 154 patients with thymic epithelial tumors were included in this study, including 80 men and 74 women, with an average age of 50.66 ± 12.45 years and an average tumor size of 6.71 ± 3.11 cm (Table 1). Table 1 also shows patient's WHO staging, T staging, smoking history, drinking history, myasthenia gravis (MG) status and other relevant clinical information.

**Table 1:** Patient and tumor-related characteristics of thymic tumor (*n* =154).

**Characteristic**	**N**	**%**
**Gender**		
Male	80	51.9
Female	74	48.1
**Age (years)**		
≤ 60	121	78.6
>60	33	21.4
**Smoking history**		
Never	119	77.3
Ever	35	22.7
**Drinking history**		
No	135	87.7
Yes	19	12.3
**Family history of tumor**		
No	131	85.1
Yes	23	14.9
**Tumor size(cm)**		
≤ 6	85	55.2
>6	69	44.8
**pT stage**		
T1	122	79.2
T2-3	32	20.8
**WHO stage**		
A-AB	62	40.3
B1-B3	77	50
C	15	9.7
**Myasthenia gravis**		
No	143	92.9
Yes	11	7.1
**ALB**		
≤ 42.6	57	37
>42.6	97	63
**A/G**		
≤ 2.0	135	87.7
>2.0	19	12.3
**BMI**		
≤ 18.8	16	10.4
>18.8	138	89.6
**HGB**		
≤ 124.0	36	23.4
>124.0	118	76.6
**NRI**		
≤ 99.6	15	9.7
>99.6	139	90.3
**NLR**		
≤ 2.7	129	83.8
>2.7	25	16.2
**PLR**		
≤ 147.9	125	81.2
>147.9	29	18.8
**SII**		
≤ 688.5	129	83.8
>688.5	25	16.2
**PNI**		
≤ 50.9	35	22.7
>50.9	119	77.3

*NLR, neutrophil-to-lymphocyte ratio; HGB, hemoglobin; ALB, albumin; A/G, albumin/globulin; BMI, body mass index; SII, systemic immune-inflammation Index; PLR, platelet-lymphocyte ratio; PNI, prognostic nutritional index; NRI, nutritional risk index; pT stage, Pathological T stage*.

### Optimal Cutoff Values for Preoperative PNI, NRI, NLR, and SII

Considering OS as the endpoint, the optimal cut-off values of preoperative PNI, NRI, NLR, and SII were determined using X-tile software. The cutoff values were as follows: PNI: 50.9 (*p* = 0.05), NRI: 99.6 (*p* = 0.000), NLR: 2.7 (*p* = 0.001), and SII: 688.5 (*p* = 0.001). For further analysis, patients were divided into low or high groups for PNI, NRI, NLR, and SII based on the relevant cut-off values.

### Association of PNI, NRI, NLR, and SII With Survival Outcomes

**Figure 1:**
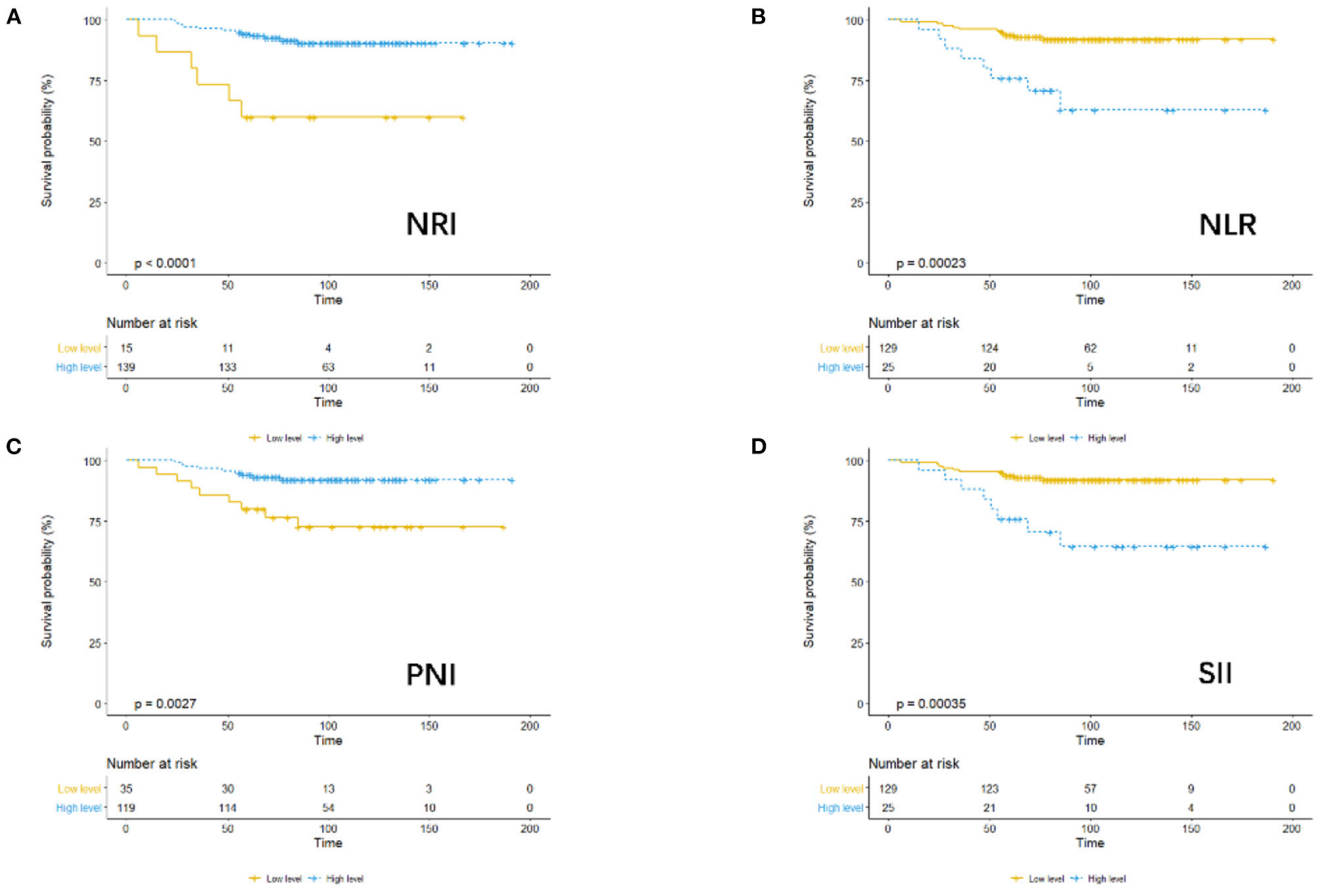
KM analysis of NRI **(A)**, NLR **(B)**, PNI **(C)**, and SII **(D)** based on overall survival.

Using OS as the endpoint, we compared the outcomes in terms of OS among patients assigned to the low- and high-level PNI, NRI, NLR, and SII groups, as demonstrated by the KM survival curves (Figure 1).

### Univariate and Multivariate Survival Analysis

According to the results of the univariate Cox regression analysis, 10 variables were significantly associated with OS: WHO stage, T stage, drinking history, BMI, ALB, PNI, NRI, NLR, SII, and A/G (Table 2). In multivariate Cox regression analysis, six parameters were defined as independent prognostic factors for OS: T stage (T1 vs. T2-3), WHO stage (A-AB vs. B1-B3, and A-AB vs. C), ALB, A/G, NRI, and NLR (Table 2).

**Table 2:** Univariate and multivariate analysis results in thymic epithelial tumor (*n* = 154).

	**Univariate analysis**		**Multivariate analysis**	
	***P***	**HR**	**95%CI**	***P***
Gender	0.079			
Male vs. Female				
Age (years)	0.939			
≤ 60 vs. >60				
Smoking history	0.275			
Never vs. Ever				
Drinking history	0.046			
No vs. Yes				
Family history of tumor	0.255			
No vs. Yes				
Tumor size	0.06			
≤ 6 vs. >6				
pT stage	0	Reference		
T1 vs. T2-3		3.542	1.118-11.228	0.032
WHO stage	0	Reference		
A-AB vs. B1-B3		0.815	0.210-3.169	
A-AB vs. C		6.699	0.1.813-24.749	0.003
Myasthenia gravis	0.418			
No vs. Yes				
ALB	0.002	Reference		
≤ 42.6 vs. >42.6		0.235	0.069-0.802	0.021
A/G	0.039	Reference		
≤ 2.0 vs. >2.0		12.182	3.178-46.693	0
BMI	0.01			
≤ 18.8 vs. >18.8				
HGB	0.097			
≤ 124.0 vs. >124.0				
NRI	0	Reference		
≤ 99.6 vs. >99.6		0.19	0.052-0.692	0.012
NLR	0.001	Reference		
≤ 2.7 vs. >2.7		3.471	1.212-9.941	0.02
PLR	0.079			
≤ 147.9 vs. >147.9				
SII	0.001			
≤ 688.5 vs. >688.5				
PNI	0.005			
≤ 50.9 vs. >50.9				

*NLR, neutrophil-to-lymphocyte ratio; HGB, hemoglobin; ALB, albumin; A/G, albumin/globulin; BMI, body mass index; SII, systemic immune-inflammation Index; PLR, platelet-lymphocyte ratio; PNI, prognostic nutritional index; NRI, nutritional risk index; pT stage, Pathological T stage*.

### coNRI-NLR Model Construction

**Figure 2:**
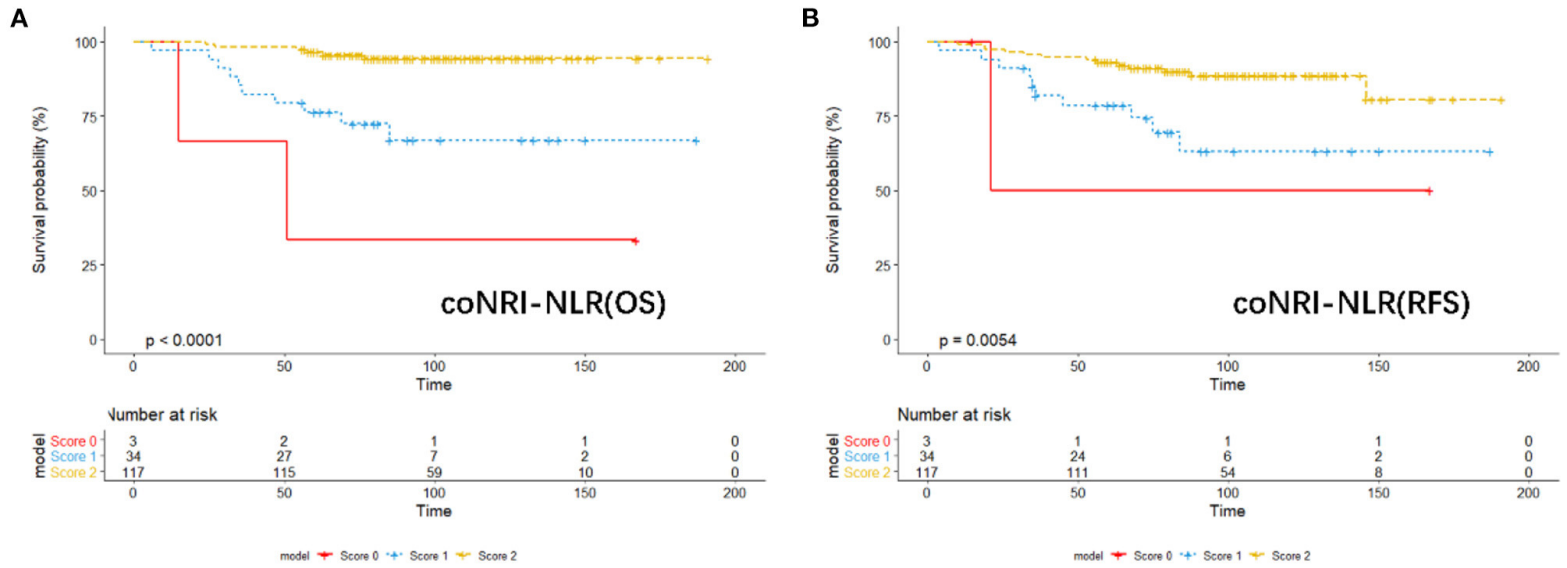
KM analysis of the model of coNRI-NLR based on overall survival **(A)** and relapse-free survival **(B)**.

**Figure 3:**
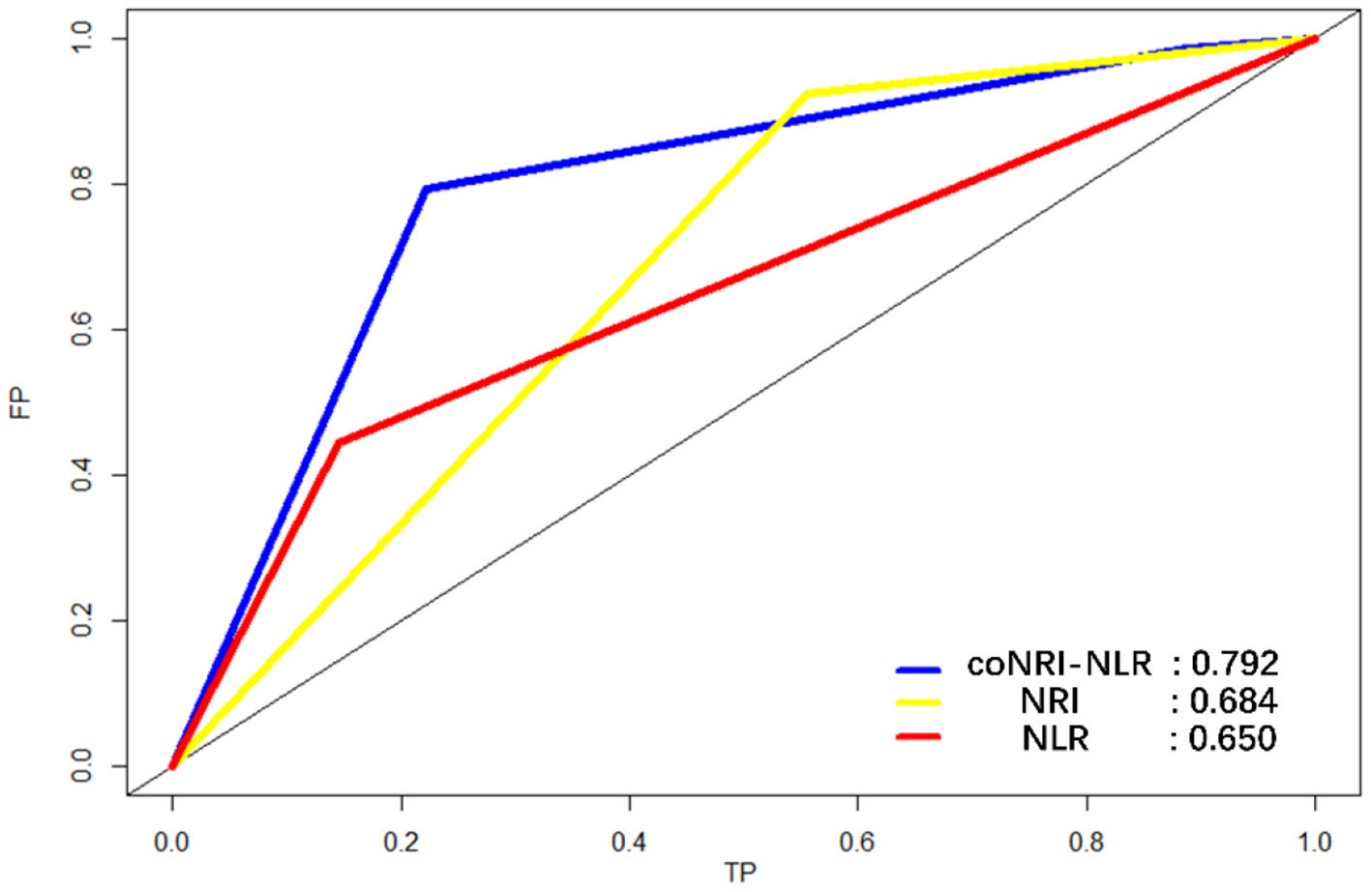
Receiver operating characteristic curve (ROC) analysis for the sensitivity and specificity of the model coNRI-NLR, NRI, and NLR.

According to the coNRI-NLR model score, those with high NRI and low NLR were given 2 points; those with high NRI and high NLR and those with low NRI and low NLR were given 1 point; and those with low NRI and high NLR were given 0 points. Patients were divided into low-risk (Score 2), middle-risk (Score 1) and high-risk (Score 0) groups and the KM curve related to OS and RFS were assessed (Figure 2; *p* < 0.001). Additionally, ROC analysis was used to compare the coNRI-NLR model with NRI and NLR. The AUC of the coNRI-NLR model value was 0.792, which was higher than that of either NRI (0.684) or NLR (0.650) alone (Figure 3).

## Discussion

By comprehensively considering multiple clinical factors and four nutritional status and immune-inflammatory indicators of patients, we conducted univariate and multivariate analyses and concluded that NRI and NLR had significant effects on OS. Additionally, the coNRI-NLR prognostic model constructed from these two factors also has the ability to predict postoperative prognosis in patients with thymic epithelial tumors.

At present, several published studies have assessed prognostic factors for patients with thymic epithelial tumors. Initially, the research of Fang et al. established a predictive model for thymic tumor recurrence through multi-center analysis combined with T staging and WHO staging (21). And a study by Luo et al. constructed a similar model by integrating lactate dehydrogenase and clinical data (22). Additionally, Wang et al. analyzed data from the Surveillance, Epidemiology, and End Results (SEER) database to establish a clinically relevant OS prognostic model (23). However, few studies have comprehensively evaluated the PNI, NRI, NLR, and SII in patients with thymic epithelial tumors.

The NLR is a hematological marker of systemic inflammation. In this study, univariate and multivariate analyses revealed that the NLR could effectively predict the OS of patients with thymic epithelial tumors. Nakajima et al. also found that elevated preoperative NLR was associated with poor prognosis after thymoma resection (24). Negri et al. also concluded that a high preoperative NLR is associated with shorter Disease Free Survival in patients undergoing thymectomy (25). In this study, the prognostic value of the NLR was better than that of the PLR, which is consistent with He's research conclusion (26). However, the current research assessing the NLR is still limited, and more patient samples and prospective studies remain to be fully evaluated.

Combining ALB and BMI, the NRI reflects the nutritional status of the body and may predict the prognosis of cancer patients. Our findings agree with a study of gastric cancer reported in 2018 (27). Subsequently, a large-scale prospective study of 1,395 patients by He et al. (10) found that the prognostic performance of the NRI was better than that of the PNI in oral cancer, which is also consistent with our findings. Furthermore, in an analysis of the preoperative immune nutritional status of 244 patients with thymoma who underwent thymectomy, Cui et al. (28) found that preoperative immune nutritional support can effectively reduce postoperative complications for thymoma patients with MG. Additionally, their intervention was found to reduce postoperative infection and the risk of complications and hospitalization.

A growing body of research has recently identified novel prognostic factors for cancer. However, most studies of this type have focused on biomarkers, which require complicated molecular and genetic testing (29, 30). The spending and complexity of these tests limit their practical application. By contrast, our study used laboratory test results as prognostic factors as part of routine clinical surveillance. In addition, blood tests routinely used in clinical medicine are more reliable than most tests performed in biological laboratories and do not require specialized equipment or expertise. As a final step in our analysis, we constructed the coNRI-NLR model which combined two independent predictors of prognosis. By comparing the area under the AUC curve, the model was found to be superior to the NRI or NLR alone in terms of its prognostic ability.

Our study proposes an efficient coNRI-NLR model that can classify patients into three subgroups with significant differences in recurrence-free survival and overall survival. It can predict the prognosis of patients with thymic epithelial tumors. The model is used as follows: if the patient has a higher NRI (>99.6) and a lower NLR (≤ 2.7) before surgery, it means that the patient may have a better prognosis, If the patient has a lower NRI (≤ 99.6) and a higher NLR (>2.7) before surgery, it is considered that the patient may have a high risk of recurrence. It is recommended that clinicians should fully evaluate the value of postoperative adjuvant therapy to implement the best possible Individualized treatment strategies.

This study has several limitations. First of all, it was a single-center study with a relatively small sample size. Secondly, our study did not analyze other important inflammatory biomarkers such as interleukin and C-reactive protein. Finally, this study did not consider postoperative dynamic changes in the related nutritional and immune-inflammatory indices.

## Conclusions

Preoperative PNI, NRI, NLR, and SII in patients with thymic epithelial tumors have prognostic value, especially NRI and NLR. Compared with other noninvasive or invasive examination methods, the values required to calculate the NRI and NLR can be obtained relatively easily and at low-cost. In addition, the coNRI-NLR model had better predictive performance than the individual indicators assessed in this study.

## Data Availability Statement

The raw data supporting the conclusions of this article will be made available by the authors, without undue reservation.

## Ethics Statement

The studies involving human participants were reviewed and approved by the Medical Ethics Committee of Sun Yat-sen University Cancer Center. Written informed consent for participation was not required for this study in accordance with the national legislation and the institutional requirements.

## Author Contributions

G-WM and Y-YH: conception and design of the work and interpretation of data. G-WM: provision of study materials or patients. Y-YH and XL: acquisition of data. S-HL, Y-YH, and YH: analysis of data. Y-YH and S-HL: drafted the manuscript. G-WM, YH, and Y-YH: substantially revised the manuscript. All authors read and approved the final manuscript.

## Funding

This work was supported by grants from the Wu Jie-Ping Medical Foundation (No. 320.6750.2020-15-7).

## Conflict of Interest

The authors declare that the research was conducted in the absence of any commercial or financial relationships that could be construed as a potential conflict of interest.

## Publisher's Note

All claims expressed in this article are solely those of the authors and do not necessarily represent those of their affiliated organizations, or those of the publisher, the editors and the reviewers. Any product that may be evaluated in this article, or claim that may be made by its manufacturer, is not guaranteed or endorsed by the publisher.

## Supplementary Material

Supplementary Figure 1 KM analysis of T stage **(A)**, WHO **(B)**, BMI **(C)**, and ALB **(D)** based on overall survival.

Supplementary Figure 2 KM analysis of A/G **(A)** and Drinking history **(B)** based on overall survival.

Supplementary Figure 3 KM analysis of T stage, WHO, BMI and ALB based on overall survival.

## Abbreviations

NLR: neutrophil-to-lymphocyte ratio
HGB: hemoglobin
ALB: albumin
A/G: albumin/globulin
BMI: body mass index
SII: systemic immune-inflammation Index
PLR: platelet-lymphocyte ratio
PNI: prognostic nutritional index
NRI: nutritional risk index
pT stage, Pathological T stage OS: overall survival
RFS: recurrence-free survival
TC: thymic carcinoma
HR: hazard ratio
CI: confidence interval
ROC: receiver operating characteristic curve
AUC: area under the curve
TET: thymic epithelial tumor.
