# Supplementary figures and images for: Prognostic Value of Preoperative Nutritional Assessment and Neutrophil-to-Lymphocyte Ratio in Patients With Thymic Epithelial Tumors
Source: Front Nutr. 2022 Jul 8;9:868336. doi: 10.3389/fnut.2022.868336 (PMC9305307; doi:10.3389/fnut.2022.868336)

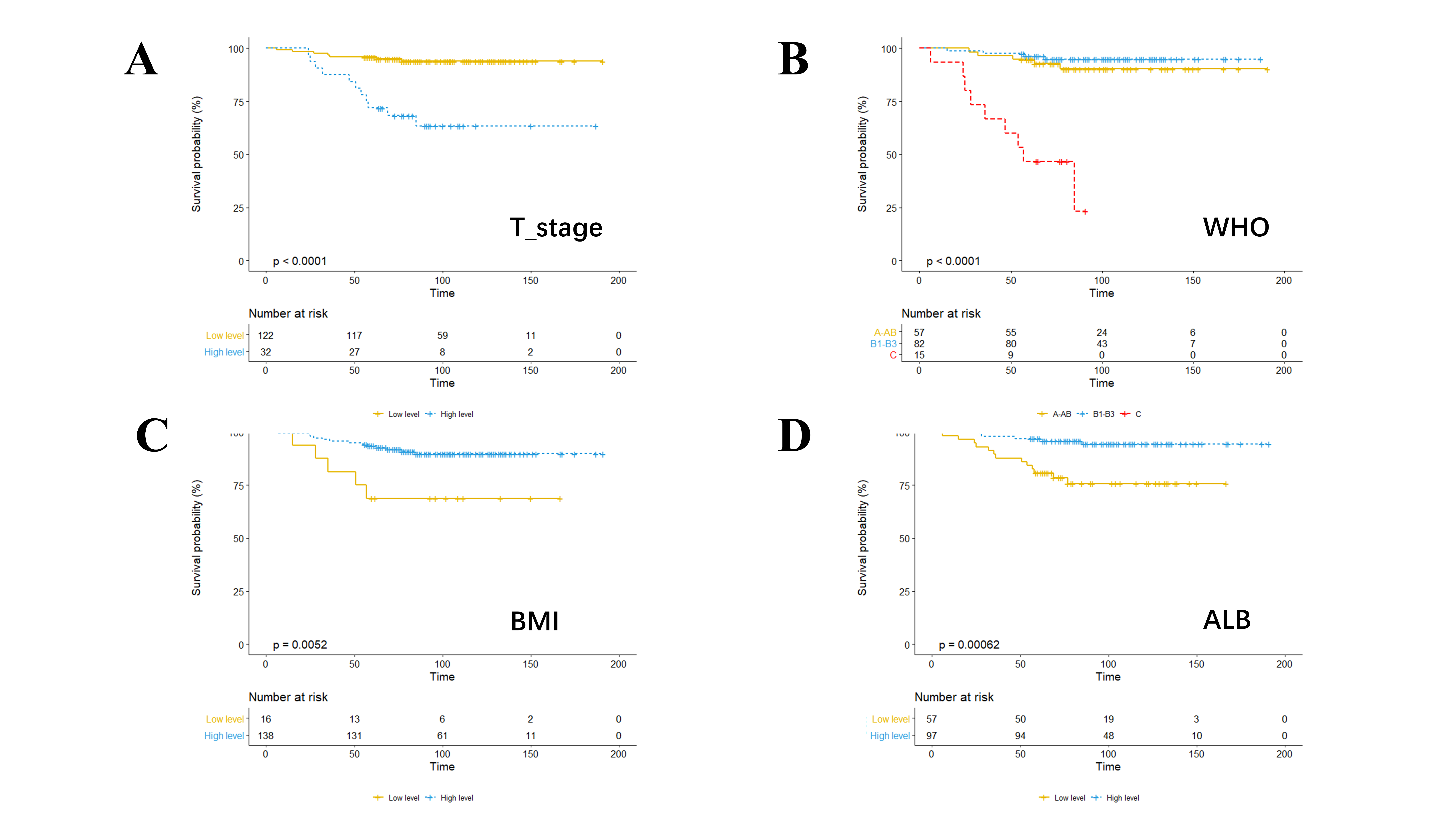

Supplement: Supplementary Figure 1 — KM analysis of T stage (A), WHO (B), BMI (C), and ALB (D) based on overall survival. [file Image_1.TIF]

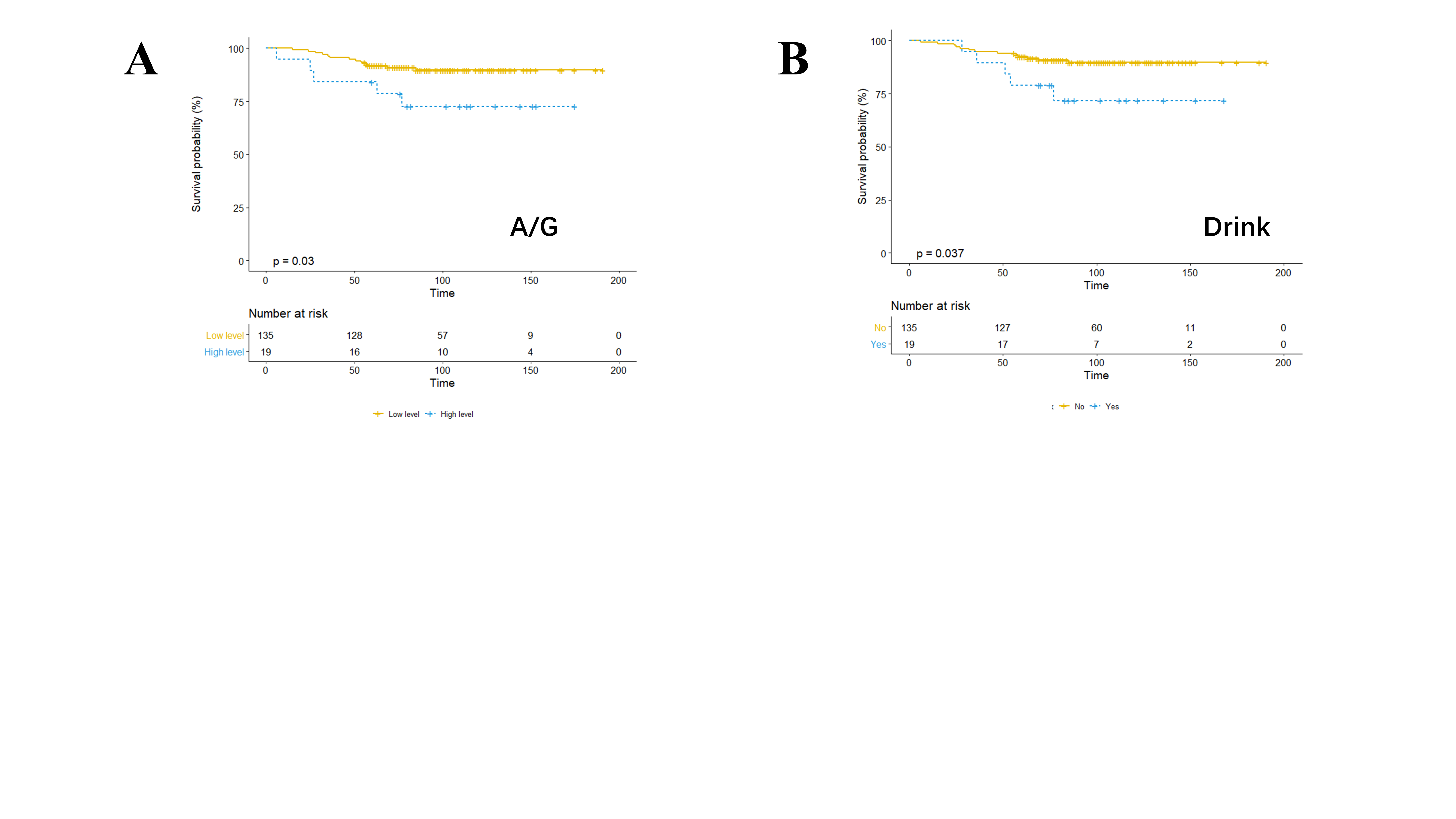

Supplement: Supplementary Figure 2 — KM analysis of A/G (A) and Drinking history (B) based on overall survival. [file Image_2.TIF]

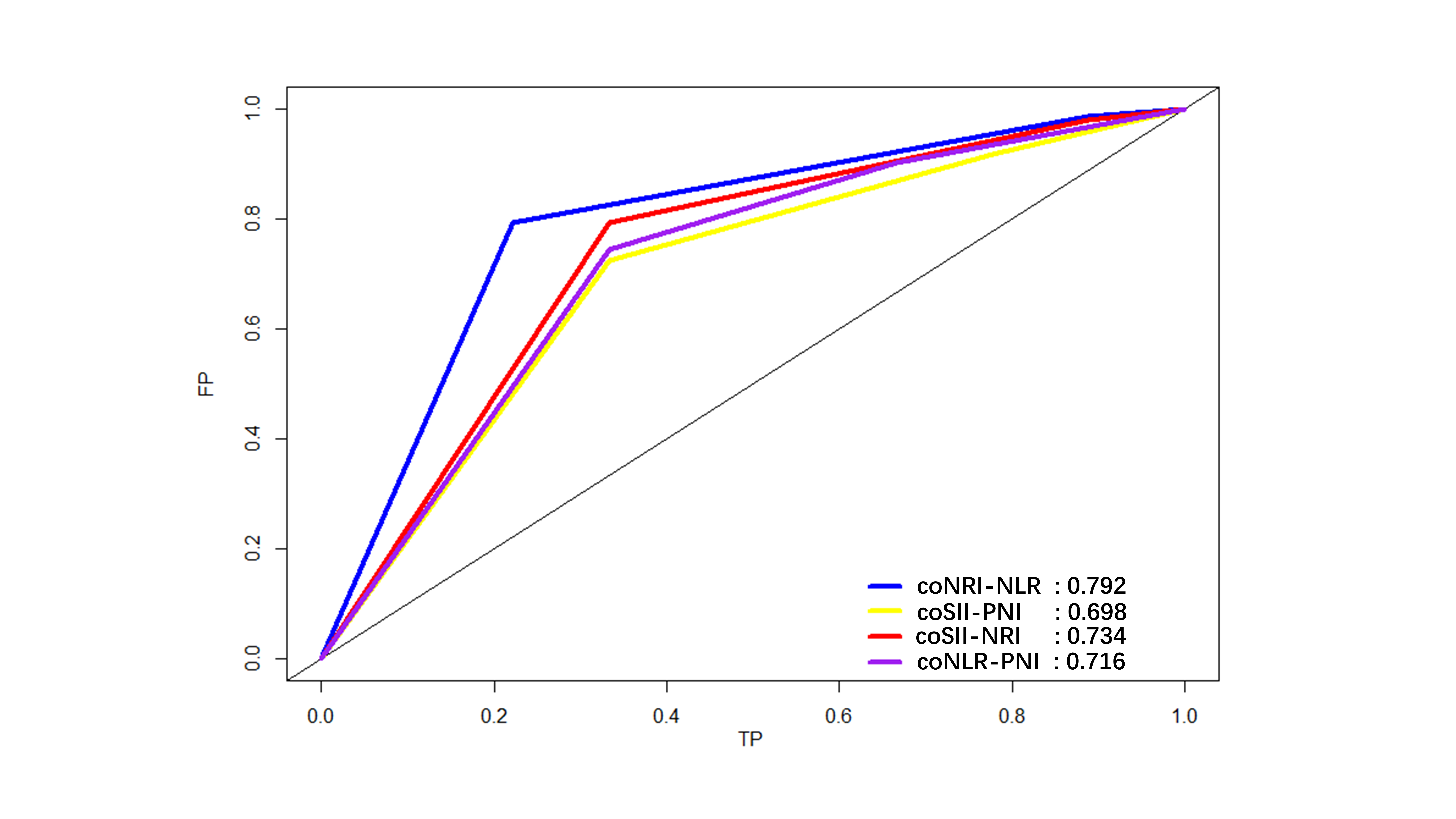

Supplement: Supplementary Figure 3 — KM analysis of T stage, WHO, BMI and ALB based on overall survival. [file Image_3.TIF]
